# A novel role of cellular interactions in vascular calcification

**DOI:** 10.1186/s12967-017-1190-z

**Published:** 2017-05-03

**Authors:** Adham Sameer A. Bardeesi, Jingwei Gao, Kun Zhang, Suntian Yu, Mengchao Wei, Pinming Liu, Hui Huang

**Affiliations:** 1 Zhongshan Medical School, Sun Yat-sen University, Guangzhou, China; 2 Guangdong Provincial Key Laboratory of Malignant Tumor Epigenetics and Gene Regulation, Department of Cardiology, Sun Yat-sen Memorial Hospital, Sun Yat-sen University, 107 West Yanjiang Road, Guangzhou, 510120 China; 3 Laboratory of RNA and Major Diseases of Brain and Heart, Sun Yat-sen Memorial Hospital, Sun Yat-sen University, Guangzhou, China

**Keywords:** Vascular calcification, Osteoblast-like cells, Autophagy, Signaling pathways

## Abstract

A series of clinical trials have confirmed the correlation between vascular calcification (VC) and cardiovascular events and mortality. However, current treatments have little effects on the regression of VC. Potent and illustrative mechanisms have been proven to exist in both bone metabolism and VC, indicating that these two processes share similarities in onset and progression. Multiple osteoblast-like cells and signaling pathways are involved in the process of VC. In this review, we summarized the roles of different osteoblast-like cells and we emphasized on how they communicated and interacted with each other using different signaling pathways. Further studies are needed to uncover the underlying mechanisms and to provide novel therapies for VC.

## Background

Vascular calcification (VC) is a pathological accumulation of calcium phosphate crystal deposits in the medial and intimal layers of the vessel wall, which complicates the course of chronic kidney disease (CKD), diabetes and atherosclerosis [1]. Patients with VC exhibit an increased risk of cardiovascular events, and no reliable treatments have been found to reverse it yet. Far from a passive process, VC is widely accepted as an active and regulatory process, which shares many similarities with the process of bone formation. Recent clinical data also indicate a close relationship between osteoporosis and VC, suggesting a crosstalk between bones and vascular systems [2]. There are a variety of osteoblast-like cells and mediators involved in this process, termed as “bone-vascular axis” [2, 3]. However, the underlying molecular mechanisms are largely unknown.

It’s widely accepted that vascular smooth muscle cells (VSMCs) have several forms of phenotypes, including contractile, osteoblastic, synthetic phenotypes, which can transfer from one to another in specific conditions. Phenotypic change of VSMCs from contractile to osteoblastic form is considered to be a possible mechanism contributing to the bone-vascular axis [4]. When exposed to pro-calcific milieu, VSMCs undergo phenotypic transition characterized by the loss of contractile markers including smooth muscle 22 alpha (SM22α) and alpha smooth muscle actin (α-SMA), followed by the increased expression of bone-related genes, such as bone morphogenetic proteins (BMPs), runt-related transcription factor 2 (Runx2), Msx, and osteocalcin [4]. There are other origins of osteoblast-like cells in the vasculature, including pericytes, endothelial cells (ECs) and circulating progenitor cells [5, 6]. Recent studies on exosomes as well as autophagy also suggest the existence of communication among these cells in VC formation [7, 8]. However, whether there are constant cellular interactions in the process of VC and the underlying signaling pathway networks are still unclear.

In the present review, we summarized the current understandings of cellular interactions in VC. We attempted to provide clinicians with in-depth insights into treating VC. Further investigations are needed to uncover the complicated interactions among these osteoblast-like cells and to achieve a breakthrough in the therapies for VC-associated diseases.

## Osteoblast-like cells in the vasculature

**Fig. 1:**
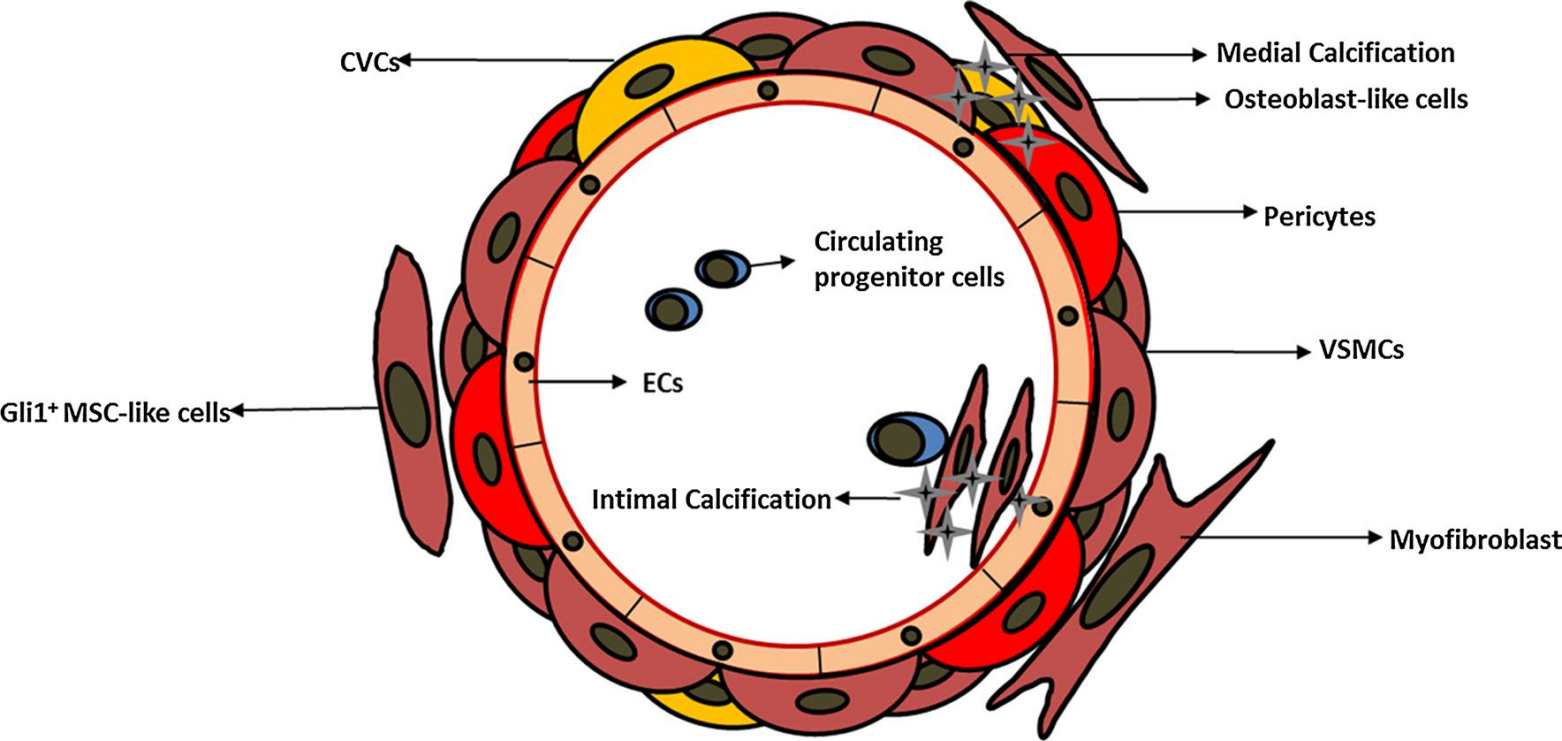
Schematic representation of osteoblast-like cells derived from different origins mediating VC. There are various origins of osteoblast-like cells in the vasculature, including ECs, pericytes, VSMCs, CVCs, myofibroblasts, and circulating progenitor cells. They collaboratively participate in the initiation and development of VC in the media or intima. *CVCs* calcifying vascular cells, *ECs* endothelial cells, *MSC* mesenchymal stem cell, *VC* vascular calcification, *VSMCs* vascular smooth muscle cells

As an active cell-mediated process, VC has been considered as bone formation by osteoblast-like cells in situ or from circulation. These cells can spontaneously produce mineralized matrix and have been identified by isolation from vascular tissue. Notably, they are derived from a variety of origins: (i) ECs in the aortic intima, (ii) pericytes in the microvessels, (iii) calcifying vascular cells (CVCs), (iv) VSMCs in the media, (v) myofibroblasts in the adventitia, and (vi) progenitor cells [5, 9] (Fig. 1).

## Endothelial cells

Endothelial-mesenchymal transition (EndMT) is a process by which ECs transit into mesenchymal stem cells and gain multipotency, prior to differentiating into various cells lineages [10]. Recent studies indicate EndMT as a key mechanism for aortic and valvular ECs to undergo osteo-/chondrogenesis [11, 12]. Usage of cinacalcet was shown to significantly abolish the up-regulation of mesenchymal markers (fibroblast-specific protein 1 and α-SMA) and the down-regulation of the endothelial marker (CD31), resulting in attenuation of VC in uremic aorta samples [13]. When stimulated with transforming growth factor-β (TGF-β), valvular ECs underwent EndMT, followed by an increase of osteocalcin, osteopontin mRNA and Runx2. Addition of valvular interstitial cells inhibited this process, indicating the importance of the interactions between valvular ECs and interstitial cells in valve calcification [14].

## Pericytes

Pericytes are perivascular cells found in abundance around ECs and share a similar support function as VSMCs. As an integral part of the microvasculature, pericytes regulate numerous functions, including vessel growth, permeability, and contractility [15, 16]. When exposed to inflammatory stimuli, pericytes turn into mesenchymal progenitors and develop into osteoblasts and chondrocytes [17]. In vivo, pericytes implanted in athymic mice were shown to develop into a variety of skeletal tissues, including bone, mineralized cartilage, and nonmineralized cartilage-like regions [18]. In long-term culture, pericytes also produce large nodules containing type I collagen, osteopontin, matrix Gla protein (MGP), and osteocalcin [19].

Clinical prescription with glucocorticoids (cortisol, corticosterone, dexamethasone) for a long period are closely associated with osteoporosis; however, these compounds have been shown to induce an osteo-/chondrogenic phenotypic switch in pericytes [20]. Elevated endogenous glucocorticoids were shown to act through mineralocorticoid receptor and facilitate phosphate-induced VSMC calcification [21]. Administration of dexamethasone reduced the expression of MGP, osteopontin and VC-associated factor mRNA on the pericytes, resulting in increased ALP activity and calcium deposition [20].

## Medial cells

The ability to undergo reversible differentiation is characteristic of the VSMC phenotype; these cells are in their differentiated and contractile form at baseline but respond to various stimuli by entering a proliferative, synthetic state. Several stimuli induce VSMCs to undergo osteogenic differentiation, including oxidative stress, inflammation or changes in pyrophosphate levels [22–24].

Growing evidence have identified the existence of a subpopulation of VSMCs and CVCs, which spontaneously form nodules and calcify when maintained in long-term culture [25, 26]. These nodules share many properties with bone, including increased alkaline phosphate (ALP) activity, osteocalcin, and osteopontin expression [27]. These cells also have the potential for multiple mesenchymal lineages, including osteoblasts, and represent 20–30% of the total VSMC population [25]. Several anti-hypertensive (spironolactone) or anti-diabetic drugs (exenatide) have been shown to exert additional effects on inhibiting CVCs calcification in vitro [28, 29].

## Adventitial cells

On the external side of the vascular wall, aortic adventitial myofibroblasts have been implicated in osteogenic programs in diabetic mice fed with high-fat diet [30]. A recent study identified Gli1^+^ mesenchymal stem cell (MSC)-like cells residing in the arterial adventitia as the progenitors of VSMCs, which contributed to neointima formation and arterial repair. However, genetic ablation of Gli1^+^ cells before injury abolishes VC [31].

## Progenitor cells

In addition to the cells surrounding the vasculature, there are several types of circulating progenitor cells participating in VC formation [32, 33]. Patients with elevated glycated hemoglobin had a higher percentage of circulating endothelial progenitor cells, which potentially mediated diabetic vasculopathy especially VC [34]. Similarly, in women with postmenopausal osteoporosis, it was demonstrated that the number of circulating osteoprogenitor cells was significantly associated with the presence and severity of abdominal aortic calcium independent of other potential confounders [35]. Bone marrow-derived and vessel-resident progenitor cells had the potential to shift into osteoblastic or osteoclastic cells in the artery wall [36]. Interestingly, treated with the peroxisome proliferator activated receptor γ agonist limited these cells to become osteoclasts instead of osteoblasts, offering a new therapeutic target for reversing VC [36].

## Signaling pathways among osteoblast-like cells

**Fig. 2:**
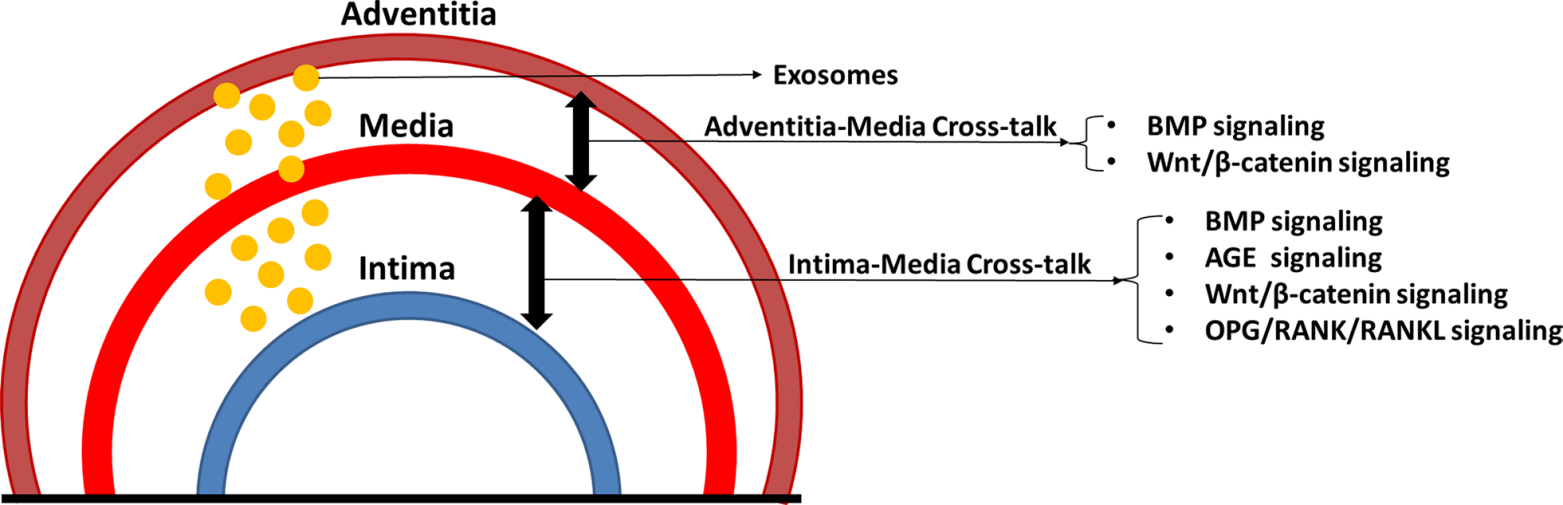
Overview of cellular interaction networks regulating calcification in the vascular wall (aortic intima, media, adventitia). During the formation of VC, there are constant cross-talks in the form exosomes among three layers of vascular wall. In addition, a complex of signaling network, including BMP, Wnt/β-catenin, AGEs and OPG/RANK/RANKL signaling pathways, are frequently seen in adventitia-media cross-talk or intima-media cross-talk. *AGEs* advanced glycation end products, *BMP* bone morphogenetic protein, *OPG* osteoprotegerin, *RANK* receptor activator of nuclear factor-kB, *RANKL* receptor activator of nuclear factor-kB ligand, *VC* vascular calcification

Although there are multiple types of osteoblast-like cells, they communicate with each other through different signaling pathways, in turn form a highly efficient network in the development of VC (Fig. 2). Thus, intervening these pathways, especially the core factors in the network, may inhibit the progression of VC. In the following sections, we will discuss a list of regulatory signaling pathways known to be involved in VC among the osteoblast-like cells.

## BMP signaling pathways

The BMPs constitute a sub-group of the TGF-β superfamily growth factors, which is essential in vascular disease. Abnormal interactions between ECs and pericytes are implicated in a number of human pathological conditions, including tumor angiogenesis, diabetic microangiopathy, and ectopic tissue calcification. Endothelium-derived TGF-β signaling is important for pericyte differentiation [37]. Similarly, in a low-density lipoprotein receptor-deficient mouse model, it was shown that MSCs in the circulation were recruited to the diseased vasculature and contributed to the development of VC. However, addition of neutralizing antibody or the inhibitor of TGF-β Receptor I kinase blocked this effect, suggesting that it was TGF-β-mediated [38]. Moreover, several BMPs activation has been associated with atherosclerosis, diabetic vasculopathy, and chronic kidney disease, all of which were known to accelerate the process of VC [39].

The BMPs have cell-specific actions and their effects on VC are dependent on the location and the type of cells they are expressed on. Aortic adventitial myofibroblasts from SM22-Cre mice secret BMP2 and participate in a pro-calcific program under high plasma glucose concentration [30]. In vascular media, BMP2 acted through the type III sodium-dependent phosphate cotransporter, Pit-1, down-regulated the microRNA-30b and 30c, resulting in an increased expression of Runx2, calcium deposition, and mineralization. The inhibition of phosphate uptake or transfection with microRNA-30b-c could abrogate these effects [40]. In addition, BMP2 elevated aggrecan message and type II collagen mRNA, which further induced osteo-/chondrogenic phenotypic switch of pericytes [41]. In diabetic mice, it was shown that BMP2 induced Msx and lipoprotein receptor-related protein (LRP) expression, and exerted pro-calcific effects [42]. Moreover, in the aortic intima, BMP4 and BMP7 had similar functions in mediating inflammation as well as EndMTs, which attenuated VC in CKD [10, 43].

Taken together, BMPs may derive from three layers of vascular wall, bind to the BMP receptors on ECs, pericytes or VSMCs, in turn accelerate medial or intimal calcification.

## OPG/RANK/RANKL signaling pathway

Recent discovery of OPG/receptor activator of nuclear factor-kB (RANK)/RANK ligand (RANKL) system builds a physiopathological link between bone loss and VC [44]. In mice, OPG knockdown presented severe osteoporosis and VC, but OPG overexpression reduced osteoclast differentiation from precursor cells and increased bone mass [45, 46]. It is clear that RANKL binds to its physiologic receptor, RANK, in turn promotes osteoclast differentiation. However, OPG interferes with the interaction between RANK and RANKL and inhibits this process [47]. It seemed inconsistent that serum OPG levels were increased in the presence of VC [48], but this may be a reflection of noxious OPG activity or a compensatory mechanism. Growing evidence have demonstrated the vascular OPG/RANK/RANKL axis plays an important role in the cellular interactions under pro-calcific setting [44].

From a total of 73 carotid plaques (49 asymptomatic and 24 symptomatic) data, Davaine et al. showed that higher presence of OPG and pericytes was significantly associated with carotid plaques stability [49]. In vitro study, further demonstrated that human vascular pericytes secreted considerable amounts of OPG and underwent osteoblastic differentiation. Pericytes also inhibited the osteoblastic differentiation of CD14+ through the secretion of OPG, suggesting the complex functions of OPG in VC [49]. Interestingly, it was demonstrated that RANKL increased BMP2 release from human aortic ECs (HAECs). The exposure of human aortic SMCs (HASMCs) to RANKL-treated HAEC-conditioned media induced osteoblastic behavior in HASMCs [50]. This phenomenon indicated the importance of HAEC-HASMC signaling to VC in the context of OPG and RANKL.

## Advanced glycation end products (AGEs) signaling pathways

Derived from non-enzymatic modification of proteins by glucose, AGEs play an important role in the pathogenesis of numerous diseases, including diabetic complications, atherosclerosis and aging [51]. AGEs formed at an accelerated rate especially under diabetes. AGEs may accumulate in heart and blood vessels, leading to accelerated plaque formation and increased cardiac fibrosis [52].

In the bovine pericyte culture, AGEs and the receptor for AGEs (RAGEs) were shown to enhance the number of calcified nodules, accompanied by an increase of ALP and OPN mRNAs [53]. Further studies demonstrated that AGEs enhanced VSMC calcification partly through a RAGEs/oxidative stress pathway [54]. Transduced HASMCs with adenovirus expressing RAGEs induced ALP, calcium deposition and osteogenic differentiation defined by an increase of Msx and BMP2 expression [55]. However, others’ and our findings indicated that administration of different antioxidants like apocynin could significantly ameliorate diabetes-induced calcification [56, 57]. Importantly, the expression of β-catenin was shown to increase with the AGEs concentrations augment in AGEs-induced VSMC calcification, suggesting that there was a cross-talk between AGEs and Wnt/β-catenin signaling pathways [58].

Thus, in diabetes, circulating AGEs may impair vascular intima, bind to RAGEs and mediate the crosstalk between ECs and VSMCs/pericytes, in turn participate in the formation of VC.

## Wnt/β-catenin signaling pathway

Wnts are a large family of conserved secreted carbohydrate and lipid-modified proteins that are involved in cell differentiation, proliferation and death [59]. They bind to cell-surface receptors of the Frizzled family and LRP-5/6, resulting in nuclear β-catenin translocation and specific gene expression [60]. Many proteins related with VC are known Wnt target genes, including Runx2 and osteocalcin. In addition to this canonical pathway, there exist β-catenin-independent pathways, such as Ca^2+^/protein kinase A pathway, G-protein/protein kinase C/JNK signaling pathway and Src/ERK pathway [61].

Glucose, inflammation and reactive oxygen species up-regulate BMP2/4 production by pericytes and ECs, in turn promote adventitial Msx2-Wnt signaling. Then, the enhanced Wnt production increased medial nuclear β-catenin accumulation, ALP activity, and osteogenic differentiation [62, 63]. The aberrant differentiation of pericytes contributed to the development and progression of several vascular pathologies [18]. Importantly, Kirton et al. demonstrated that canonical Wnt/β-catenin signaling could inhibit adipogeic differentiation and enhance chondrogenic differentiation of pericytes [64]. Two vitamin D receptor agonists, calcitriol and paricalcitol have differential effects on VC, which was mediated by a distinct regulation of the BMP and Wnt/β-catenin signaling pathways [65]. Similarly, activation of Wnt/beta-catenin signaling pathway was also shown to motivate calcium deposition via BMP-2 in pericytes, indicating that these two pathways may interact synergistically to contribute VC [66].

However, the role of non-canonical Wnts pathways in the process of VC is still under debate. It was shown that LRP6 promoted osteo-/chondrogenic differentiation of VSMCs via upstream stimulatory factor1- and arginine methylation-dependent relays [67]. But in calcified aortic valves, Albanese et al. found a significant correlation between Wnt5b and Wnt11 overall staining and the presence of calcification, which could be reduced by mitogen-activated protein kinase-38β and GSK3 β inhibitors [68]. These contradictory results could be attributed to bidirectional functions of non-canonical Wnts pathways in different cells lineages.

Like BMP signaling pathway, Wnt/β-catenin signaling also plays an important role in the crosstalk among aortic intima, media, adventitia.

## Other evidence of cellular interactions in VC

In addition to multiple signaling pathways, there are also constant interactions in terms of ‘message carriers’ among osteoblast-like cells (Fig. 2). Outside disease milieu, uremic serum from patients was shown to induce calcification of cultured VSMCs, suggesting the pro-calcific messengers in CKD serum, termed as exosomes [69]. Exosomes are novel intercellular communication messengers containing biologically active proteins, lipids and RNA species. Pro-inflammatory cytokines and growth factors, as well as mineral imbalance, stimulate VSMCs to secrete thousands of exosomes. They play important roles in vascular repair, thrombosis and calcification in a paracrine or autocrine manner [70]. In response to environmental calcium stress, VSMCs can autonomously secret multiple exosomes enriched with sphingomyelin phosphodiesterase 3 and CD63, leading to increased calcification of neighboring normal VSMCs [8].

In addition, autophagy is an adaptive stress response whereby cells remove unnecessary or dysfunctional cellular components by targeting them to modified lysosomes for degradation or recycling [7]. Emerging evidence indicate that autophagy also regulates extracellular matrix homeostasis and mitigates VC formation [71, 72]. In the setting of hyperphosphatemia, autophagy may be an endogenous protective mechanism counteracting phosphate-induced VC. Administration of an inducer of autophagy, valproic acid, significantly decreased VC. However, pretreatment of an autophagy inhibitor, 3-methylademine, promoted matrix vesicle release with increased ALP activity [73].

Except for VSMCs, other types of vascular cells may also have the potential to secrete exosomes under pro-calcific conditions, and influence nearby normal cells and accelerate VC formation.

## Conclusion

Vascular calcification is a hallmark and major risk factor for cardiovascular morbidity and mortality. In this present review, we highlight the findings that multiple osteoblast-like cells and signaling pathways are involved in the initiation and development of VC. However, direct evidence about the roles of cellular interactions in VC was limited and the underlying mechanisms are still not very clear. Thus, future studies are required to explore these mechanisms in detail. With more clues uncovered, comprehensive treatments targeting cellular interactive network may provide clinicians with a novel therapy for attenuating VC and reducing cardiovascular events.

## Abbreviations

AGEs: advanced glycation end-products
α-SMA: alpha smooth muscle actin
RAGEs: receptor for advanced glycation end products
ALP: alkaline phosphatase
BMPs: bone morphological proteins
CKD: chronic kidney disease
CVCs: calcifying vascular cells
ECs: endothelial cells
EndMT: endothelial-mesenchymal transition
HAECs: human aortic endothelial cells
HASMCs: human aortic smooth muscle cells
LRP: lipoprotein receptor-related protein
MGP: matrix Gla protein
MSC: mesenchymal stem cell
OPG: osteoprotegerin
RANK: receptor activator of nuclear factor-kB
RANKL: receptor activator of nuclear factor-κB ligand
SM22α: smooth muscle 22 alpha
TGF-β: transforming growth factor-β
VSMCs: vascular smooth muscle cells
VC: vascular calcification
